# Tricyclic Guanidine Alkaloids from the Marine Sponge *Acanthella cavernosa* that Stabilize the Tumor Suppressor PDCD4

**DOI:** 10.3390/md12084593

**Published:** 2014-08-21

**Authors:** Tanja Grkovic, Johanna S. Blees, Magdalena M. Bayer, Nancy H. Colburn, Cheryl L. Thomas, Curtis J. Henrich, Megan L. Peach, James B. McMahon, Tobias Schmid, Kirk R. Gustafson

**Affiliations:** 1 Molecular Targets Laboratory, Center for Cancer Research, National Cancer Institute, Frederick, MD 21702, USA; E-Mails: t.grkovic@griffith.edu.au (T.G.); johanna.blees@gmail.com (J.S.B.); cltterry@mail.nih.gov (C.L.T.); henrichcj@mail.nih.gov (C.J.H.); mcmahoja@mail.nih.gov (J.B.M.); 2 Institute of Biochemistry I, Faculty of Medicine, Goethe-University Frankfurt, 60590 Frankfurt, Germany; E-Mail: M_Bajer@gmx.de; 3 Laboratory of Cancer Prevention, Center for Cancer Research, National Cancer Institute, Frederick, MD 21702, USA; E-Mail: colburna@mail.nih.gov; 4 Basic Science Program, Leidos Biomedical Research, Inc., Frederick National Laboratory for Cancer Research, Frederick, MD 21702, USA; 5 Basic Science Program, Leidos Biomedical Research, Inc., Chemical Biology Laboratory, Frederick National Laboratory for Cancer Research, Frederick, MD 21702, USA; E-Mail: mpeach@helix.nih.gov

**Keywords:** natural product, guanidine alkaloid, PDCD4, tumor suppressor

## Abstract

A cell-based high-throughput screen that assessed the cellular stability of a tumor suppressor protein PDCD4 (Programmed cell death **4**) was used to identify a new guanidine-containing marine alkaloid mirabilin K (**3**), as well as the known compounds mirabilin G (**1**) and netamine M (**2**). The structures of these tricyclic guanidine alkaloids were established from extensive spectroscopic analyses. Compounds **1** and **2** inhibited cellular degradation of PDCD4 with EC_50_ values of 1.8 μg/mL and 2.8 μg/mL, respectively. Mirabilin G (**1**) and netamine M (**2**) are the first marine natural products reported to stabilize PDCD4 under tumor promoting conditions.

## 1. Introduction

**Figure 1:**
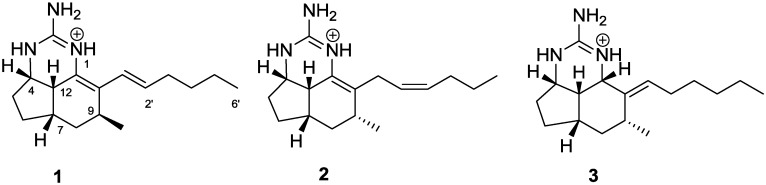
Structures of the tricyclic guanidine alkaloids mirabilin G (**1**), netamine M (**2**), and mirabilin K (**3**).

Tumor suppressor proteins can prevent or repress malignant cell growth by regulating progression of the cell cycle or by promoting apoptosis via alteration in gene expression patterns. Rather than directly affecting transcription, PDCD4 (programmed cell death **4**) is a novel tumor suppressor that inhibits protein translation through interaction with the eukaryotic translation initiation factors eIF4A and eIF4G [1]. PDCD4 can inhibit the transformation, migration, and invasion of cancer cells *in vitro* [2,3,4], and overexpression of PDCD4 in transgenic mice was found to significantly suppress tumorigenesis [5]. Down regulation of PDCD4 expression has been associated with the onset of a number of human tumors including colorectal [6], brain [7], ovarian [8], and liver carcinomas [9]. Since cellular PDCD4 levels are in part regulated by phosphorylation-dependent proteasomal degradation in response to tumor promoters [10,11,12], stabilizing PDCD4 provides an attractive potential therapeutic target. With the aim to identify small molecule stabilizers of PDCD4, a high throughput cell-based reporter screen was developed where the stability of PDCD4 was assessed under tumor promoting conditions [13]. The assay was designed to monitor tetradecanoylphorbol-13-acetate (TPA)-induced degradation of a PDCD4-luciferase construct, and a 50% or greater recovery of the luciferase signal was defined as a hit. The screening assay was used to test natural product extracts sourced from a diverse collection of marine invertebrates, terrestrial plants, and microbial isolates from the Natural Products Repository of the US National Cancer Institute. Testing of 135,678 extracts yielded 42 confirmed hits to date. Previously we reported on the PDCD4 stabilizing activity of tubercidin from a halophilic actinomycete *Actinopolyspora erythrea* [14], as well as terrestrial plant metabolites including the cryptocaryols, a series of α-pyrone-containing 1,3 polyols obtained from *Cryptocarya* sp. [15]; erioflorin, a sesquiterpene lactone isolated from the wooly sunflower *Eriophyllum lanatum* [16], and the isoflavone pomiferin triacetate [17]. Herein we report on the activity of compounds isolated from the organic solvent extract of the marine sponge *Acanthella cavernosa* (NSC # C005465), collected in Southwestern Australia. Bioassay-guided fractionation resulted in the purification of two known compounds, mirabilin G (**1**) [18,19] and netamine M (**2**) [20], along with a new analogue, mirabilin K (**3**), as shown in Figure 1. These metabolites belong to a class of tricyclic guanidine alkaloids exemplified by the ptilocaulins, [21,22,23] netamines, [20,24] and mirabilins [18,19,25].

## 2. Results and Discussion

An aliquot of the crude CH_2_Cl_2_/MeOH extract (170 mg) of the sponge was subjected to diol flash chromatography eluting sequentially with hexanes, CH_2_Cl_2_, EtOAc, and MeOH. The PDCD4 stabilizing activity concentrated in the MeOH fraction, which was further subjected to C_18_, C_8_, and C_2_ reversed-phase chromatography. The trifluoroacetate salt of mirabilin G (**1**, 2.4 mg, 1.41% crude weight) was purified using a flash C_8_ reversed-phase column, while the trifluoroacetate salt of mirabilin K (**3**, 0.8 mg, 0.47% crude weight) was purified by C_2_ semipreparative HPLC. Each chromatography fraction was monitored by LCMS and ^1^H NMR, and it was apparent that signals corresponding to an additional analogue were lost with the addition of aqueous TFA to the chromatography solvents. The isolation was subsequently repeated without TFA using 373 mg of extract, with the PDCD4 stabilizing activity again concentrated in the MeOH eluent from the diol flash column. This fraction was then further purified by flash C_8_ reversed-phase chromatography to yield mirabilin G (**1**, 7.4 mg, 1.98% crude weight), netamine M (**2**, 4.8 mg, 1.29% crude weight) and an impure fraction that by MS and NMR analysis contained mirabilin K (**3**).

**Figure 2:**
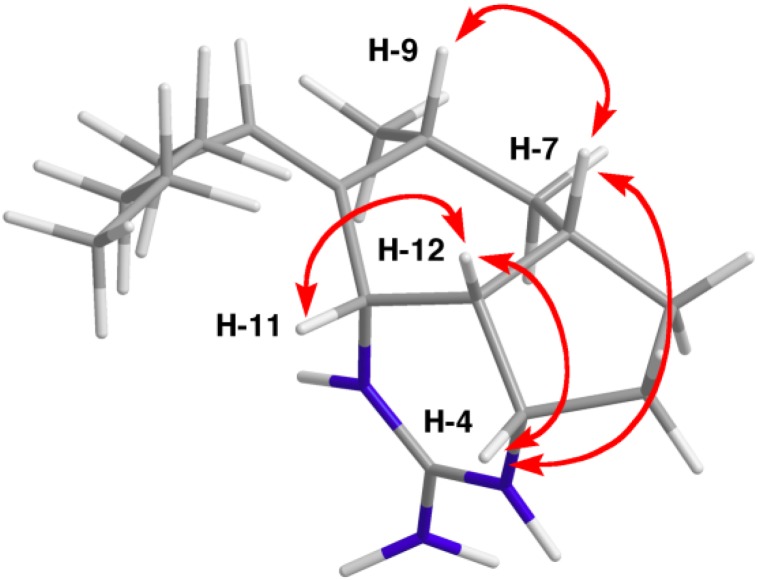
Chem3D minimized core structure of mirabilin K (**3**) showing crucial ROESY correlations used to assign the relative configuration (4S*,7S*,9R*,11S*12R*).

Mirabilin G trifluoroacetate salt (**1**) and netamine M naturally occurring counterion (**2**) were identified by comparison of their spectroscopic and chirooptical data with published values [18,19,20]. HRESIMS data for compound **3** established a molecular formula of C_17_H_30_N_3_, which required 5 double bond equivalents. Examination of the ^1^H and ^13^C NMR data for **3** revealed close correspondence to the NMR data for compound **1** (see Supplementary Table S1). However, compound **3** had only 3 sp^2^ hybridized carbon resonances which were characteristic of a guanidine group (δ_C_ 154.0) and a trisubstituted olefin (δ_C_ 138.1 and δ_C_ 132.2/δ_H_ 5.42). Another significant difference between **1** and **3** was the presence of a second nitrogen-substituted methine group (δ_H_ 3.98, d, *J* = 6.3 Hz, δ_C_ 54.9). This doublet methine showed HMBC correlations to C-2 (δ_C_ 154.0), C-7 (δ_C_ 34.6), C-9 (δ_C_ 30.0), C-12 (δ_C_ 39.6), and the two olefinic carbons, which placed it at C-11. The double bond was positioned between C-10 and C-1′ to complete the structure of mirabilin K (**3**). The relative configuration of **3** was assigned from ROESY data and coupling constant analysis. ROESY correlations from H-4 to both H-7 and H-12, H-7 to H-9, and H-11 to H-12 (see Table 1 and Figure 2) positioned H-4, H-7, H-9, H-11 and H-12 on a common face of the molecule. The 9-Me-*anti* assignment was further confirmed by analysis of the coupling constants of the resonances assigned to H-8α and β. Observation of a strong ROESY correlation from H-7 to H-8β (δ_H_ 1.84, ddd, *J* = 13.5, 5.0, 5.0 Hz) placed this resonance on the β-face of the molecule, while the coupling constant values observed for H-8α (δ_H_ 1.10, ddd, *J* = 13.5, 12.6, 12.6 Hz) confirmed it had a *trans*-di-axial relationship with both the H-7 and H-9 protons. The relative configuration of mirabilin K (**3**) was therefore proposed to be (4S*,7S*,9R*,11S*12R*).

**Table 1:** NMR data for mirabilin K (**3**) trifluoroacetate salt in CDCl_3_.

Position	δ_C_^a^_,_ Type	δ_H_, Mult. (*J* in Hz) ^b^	HMBC ^c^	ROESY
1-*N*		7.71, br s		
2	154.0, C			
C2-*N*H_2_		7.00, br s		
3-*N*		7.05, br s		
4	52.9, CH	3.80, br t (3.9)	2, 6, 7	5b, 7, 12
5a	32.9, CH_2_	1.89, m	4, 7, 12	
5b		1.64, m	4, 7, 12	4
6a	29.6, CH_2_	1.99, m	4, 5, 12	
6b		1.43, m	4, 5, 12	
7	34.6, CH	2.05, m	4,6,12	4, 8a, 9
8a	37.2, CH_2_	1.84, ddd (13.5, 5.0, 5.0)	7, 9, 9Me, 12	7, 8b, 9
8b		1.10, ddd (13.5, 12.6, 12.6)	6, 9, 9Me, 12	8a
9	30.0, CH	2.50, m	8, 10, 11, 1′	7
9-Me	22.4, CH_3_	1.06 3H, d (6.8)	8, 9, 10	8a
10	138.1, C			
11	54.9, CH	3.98, d (6.3)	7, 9, 10, 12, 1′	1′, 12
12	39.6, CH	2.31, m	4, 7, 8, 11	4, 11
1′	132.2, CH	5.42, br t (7.1)	9, 11, 3′	11, 2′, 3′
2′	27.8 CH_2_	2.02, m	1, 10, 3′	
3′	29.6, CH_2_	1.24, m	4′, 5′	
4′	31.5, CH_2_	1.26, m	3′, 5′	
5′	22.5, CH_2_	1.28, m	6′, 4′	
6′	14.0, CH_3_	0.88, t (6.8)	4′, 5′	

^a^ Recorded at 150 MHz; ^b^ Recorded at 600 MHz; ^c^ Optimized for 8.3 Hz, correlations are from the proton(s) stated to the indicated carbon.

Mirabilin G (**1**) and netamine M (**2**) were found to inhibit TPA-induced degradation of PDCD4 with EC_50_ values of 1.8 and 2.8 μg/mL, respectively, while mirabilin K (**3**) was inactive at the highest test concentration of 20 μg/mL (Table 2). Compound **2** was found to have a broader PDCD4 stabilizing range, exhibiting approximately 90% recovery of the luciferase signal at 5.5 μg/mL and cytotoxicity observed at 17 μg/mL. In contrast, mirabilin G (**1**) showed a maximum of 60% luciferase recovery and it was found to be cytotoxic at a significantly lower concentration of 4.2 μg/mL.

**Table 2:** Biological activities of compounds **1**–**3**.

Compound	PDCD4 EC_50_^a^ (μg/mL)	PDCD4 EC_90_^a^ (μg/mL)	Cytotoxicity ^b^ (μg/mL)
**1**	1.8	-	4.2
**2**	2.8	5.5	17.0
**3**	n.a. ^c^	-	10.6
Rapamycin ^d^	0.02	0.1	>1.0

^a^ EC_50_ and EC_90_ values represent the minimum concentration required for 50% and 90% recovery of the PDCD4-luciferase signal from TPA-induced degradation; ^b^ Values presented as the minimum concentration required for total luciferase signal loss, relative to TPA treated control cells; ^c^ Not active; ^d^ Positive control, concentration in μM.

## 3. Experimental Section

### 3.1. General Experimental Procedures

Optical rotations were recorded on a PerkinElmer 241 Polarimeter (PerkinElmer, Waltham, MA, USA) using a 1 dm cell in the solvent indicated. Ultraviolet-visible spectra were run as methanol solutions on a Varian Cary 50-Bio UV-Vis scanning spectrophotometer (Varian, Palo Alto, CA, USA). NMR spectra were recorded on a Bruker Avance DRX-600 spectrometer (Bruker BioSpin, Billerica, MA, USA) operating at 600 MHz for ^1^H nuclei and 150 MHz for ^13^C nuclei. Residual solvent signals were used as reference: CDCl_3_ δ_H_ 7.25, δ_C_ 77.0. Standard Bruker pulse sequences were utilized. High-resolution mass spectra were recorded using the Agilent 1100 Series LC/MSD model G1946D, with an API-electrospray source (Agilent Technologies, Santa Clara, CA, USA). Semi-preparative reversed-phase HPLC was run on a Varian ProStar 215 HPLC system (Varian, Palo Alto, CA, USA) using a Chromanetics Lichosorb C_2_ column (310 Å; 250 × 10 mm, Chromanetics Scientific Products, Trenton, NJ, USA) and eluting with a linear gradient of H_2_O (0.05% TFA) to MeCN at 2.0 mL/min. Reversed-phase flash column chromatography was carried out on C_8_ YMC stationary support with a pore size of 40–63 μm. Normal phase flash chromatography was carried out on Applied Separations Spe-ed SPE 2g diol cartridges (Applied Separations, Allentown, PA, USA).

### 3.2. Extraction and Isolation

The sponge sample was repeatedly extracted with CH_2_Cl_2_–MeOH (1:1) and 100% MeOH according to the methodology described by McCloud [26] to give the organic solvent crude extract. A portion of the crude organic extract (170 mg) was subjected to diol flash chromatography eluting with hexanes, CH_2_Cl_2_, EtOAc and MeOH. Mirabilin G (**1**) trifluoroacetate salt (2.4 mg, 1.41% crude weight) was purified from the MeOH fraction using a flash C_8_ reversed-phase column, eluting with MeOH–aqueous TFA (0.05%) (7:3). Mirabilin K (**3**) trifluoroacetate salt (0.8 mg, 0.47% crude weight) was purified from the MeOH diol fraction by semipreparative HPLC using a C_2_ reversed-phase column, eluting with MeCN–aqueous TFA (0.05%) (7:3), at 2 mL/min. Another portion of the crude extract (373 mg) was subjected to diol flash chromatography eluting with hexanes, CH_2_Cl_2_, EtOAc and MeOH, with the activity again concentrated in the MeOH fraction. The active fraction was further subjected to flash C_8_ reversed-phase chromatography eluting with MeOH-H_2_O (6:4) to yield mirabilin G (**1**) (7.4 mg, 1.98% crude weight) and mirabilin K (**2**) (4.8 mg, 1.29% crude weight).

Mirabilin G (**1**) naturally-occurring counterion: clear oil; 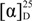 = +170 (*c* 0.1, CHCl_3_); UV (MeOH) λ (log ε) 202 (4.09), 260 (4.10) nm; ^1^H NMR (CHCl_3_, 600 MHz) and ^13^C NMR (CDCl_3_, 150 MHz) data, see Supplementary Table S1; HRESIMS *m/z* [M + H]^+^ 274.2377 (calcd for C_17_H_28_N_3_, 274.2278). Trifluoroacetate salt: yellow oil; 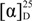 = +64 (*c* 0.325, CHCl_3_); HRESIMS *m/z* [M]^+^ 274.2270 (calcd for C_17_H_28_N_3_, 274.2278).

Netamine M (2) naturally-occurring counterion: clear oil; 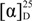 = +60 (*c* 0.1, CHCl_3_); UV (MeOH) λ (log ε) 202 (4.20), 230 (3.89) nm; ECD (MeOH) λ_max_ (Δε) 204 (−2.9), 207 (0), 253 (0), 231 (+8.2), 288 (0) nm; ^1^H NMR (CHCl_3_, 600 MHz) and ^13^C NMR (CDCl_3_, 150 MHz) data, see Supplementary Table S1; HRESIMS *m/z* [M]^+^ 274.2274 (calcd for C_17_H_28_N_3_, 274.2278).

Mirabilin K (**3**) trifluoroacetate salt: yellow oil; 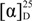 = +40 (*c* 0.1, CHCl_3_); UV (MeOH) λ (log ε) 202 (4.07), 240 (3.54) nm; ^1^H NMR (CDCl_3_, 600 MHz) and ^13^C NMR (CDCl_3_, 150 MHz) data, see Table 1; HRESIMS *m/z* [M]^+^ 276.2439 (calcd for C_17_H_30_N_3_, 276.2434).

### 3.3. PDCD4 Assay

Stabilization of PDCD4 was assessed as previously described [14], using HEK-293 cells that could be readily transfected with appropriate PDCD4-luciferase plasmids. In brief, HEK-293 renal cells expressing a fusion protein comprised of a fragment of PDCD4 containing the stability regulatory region (amino acids 39–91) and luciferase were plated (2000 cells/well, 40 μL/well) in 384-well opaque white plates and allowed to attach overnight (18 h). TPA (final concentration 10 nM) was added followed (within 15 min) by test samples or controls. Following an 8 h incubation, luciferase activity was measured 10–15 min after the addition of Steadylite Plus (Perkin-Elmer) reagent. Controls were DMSO only (no TPA), TPA only, and TPA + rapamycin (100 nM final concentration). The activities of compounds were calculated using the following formula: activity (%) = (RLU_compound+TPA_ − RLU_TPA_)/(RLU_DMSO_ − RLU_TPA_) × 100. Cytotoxicity was estimated based on the loss of luciferase activity in cells treated with TPA and the test compound relative to control cells treated only with TPA.

## 4. Conclusions

Using a high-throughput screen to identify natural products with the ability to stabilize the tumor suppressor protein PDCD4, we isolated three tricyclic guanidine alkaloids from an extract of the Australian marine sponge *Acanthella cavernosa.* The known compounds, mirabilin G (**1**) and netamine M (**2**), were found to be the active PDCD4 stabilizing compounds in the extract, while the structural analogue, mirabilin K (**3**), had a new structure but was inactive. Compounds that prevent PDCD4 degradation have attracted considerable attention recently as evidenced by efforts directed toward the total synthesis and SAR studies of the cryptocaryols [27,28,29]. To the best of our knowledge, this is the first report of a structural class of marine alkaloids that can rescue cellular PDCD4 levels under tumor-promoting conditions.

## Supplementary Files

Supplementary File 1
