# Supplementary material for: Tricyclic Guanidine Alkaloids from the Marine Sponge Acanthella cavernosa that Stabilize the Tumor Suppressor PDCD4
Source: Mar Drugs. 2014 Aug 21;12(8):4593–601. doi: 10.3390/md12084593 (PMC4145332; doi:10.3390/md12084593)
Supplement: Supplementary File 1 [file marinedrugs-12-04593-s001.pdf]

Supplementary Information

Figure S1. ^{1}H NMR spectrum of mirabilin G (**1**) naturally-occurring counterion in $CDCl_3$ S2
Figure S2. ^{1}H NMR spectrum of mirabilin G (**1**) naturally-occurring counterion in $CDCl_3$ S3
Figure S3. ^{1}H NMR spectrum of netamine M (**2**) naturally-occurring counterion in $CDCl_3$ S4
Figure S4. ^{13}C NMR spectrum of netamine M (**2**) naturally-occurring counterion in $CDCl_3$ S5
Figure S5. ^{1}H NMR spectrum of mirabilin K (**3**) trifluoroacetate salt in $CDCl_3$ S6
Figure S6. ^{13}C NMR spectrum of mirabilin K (**3**) trifluoroacetate salt in $CDCl_3$ S7
Figure S7. ROESY spectrum of mirabilin K (**3**) trifluoroacetate salt in $CDCl_3$ S8
Figure S8. HRESIMS spectrum of mirabilin K (**3**) trifluoroacetate salt S9
Table S1. ^{1}H (600 MHz) and ^{13}C (150 MHz) data in $CDCl_3$ for mirabilin G (**1**) naturally-occurring counterion, netamine M (**2**) naturally-occurring counterion, and mirabilin K (**3**) trifluoroacetate salt S10

Figure S1. ^{1}H NMR spectrum of mirabilin G (**1**) naturally-occurring counterion in $CDCl_3$.

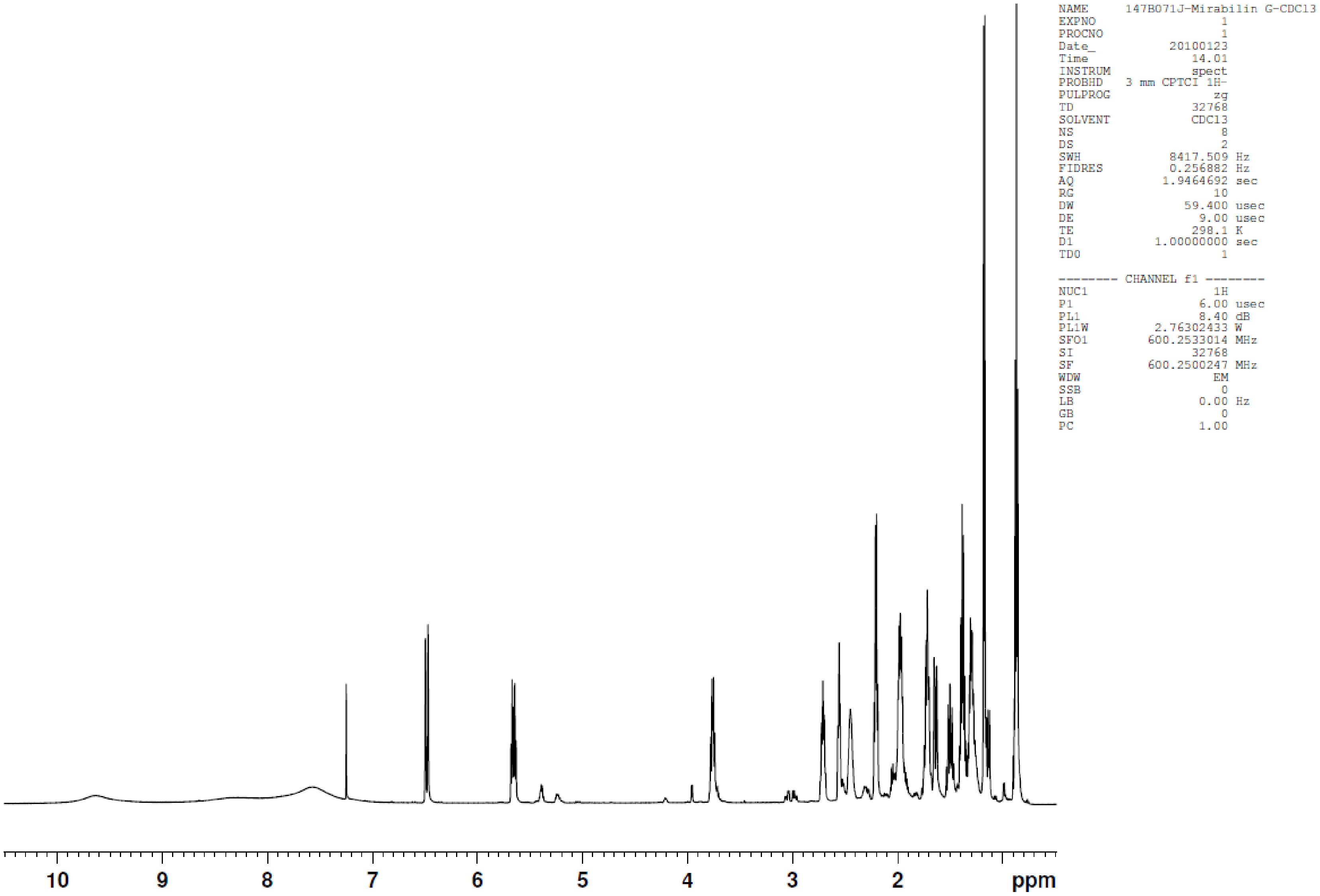

Figure S2. ^{13}C NMR spectrum of mirabilin G (**1**) naturally-occurring counterion in $CDCl_3$.

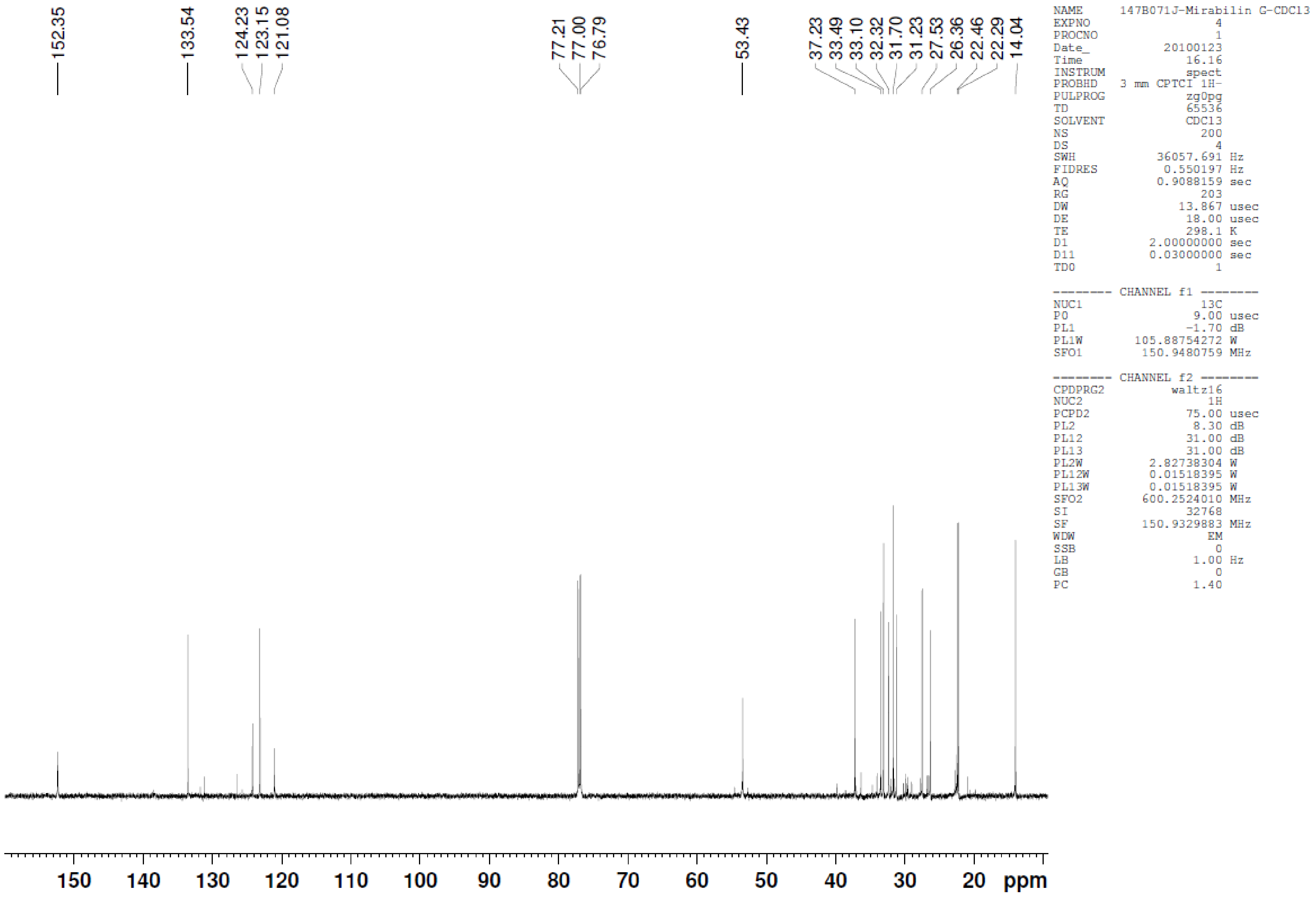

Figure S3. ^{1}H NMR spectrum of netamine M (**2**) naturally-occurring counterion in $CDCl_3$.

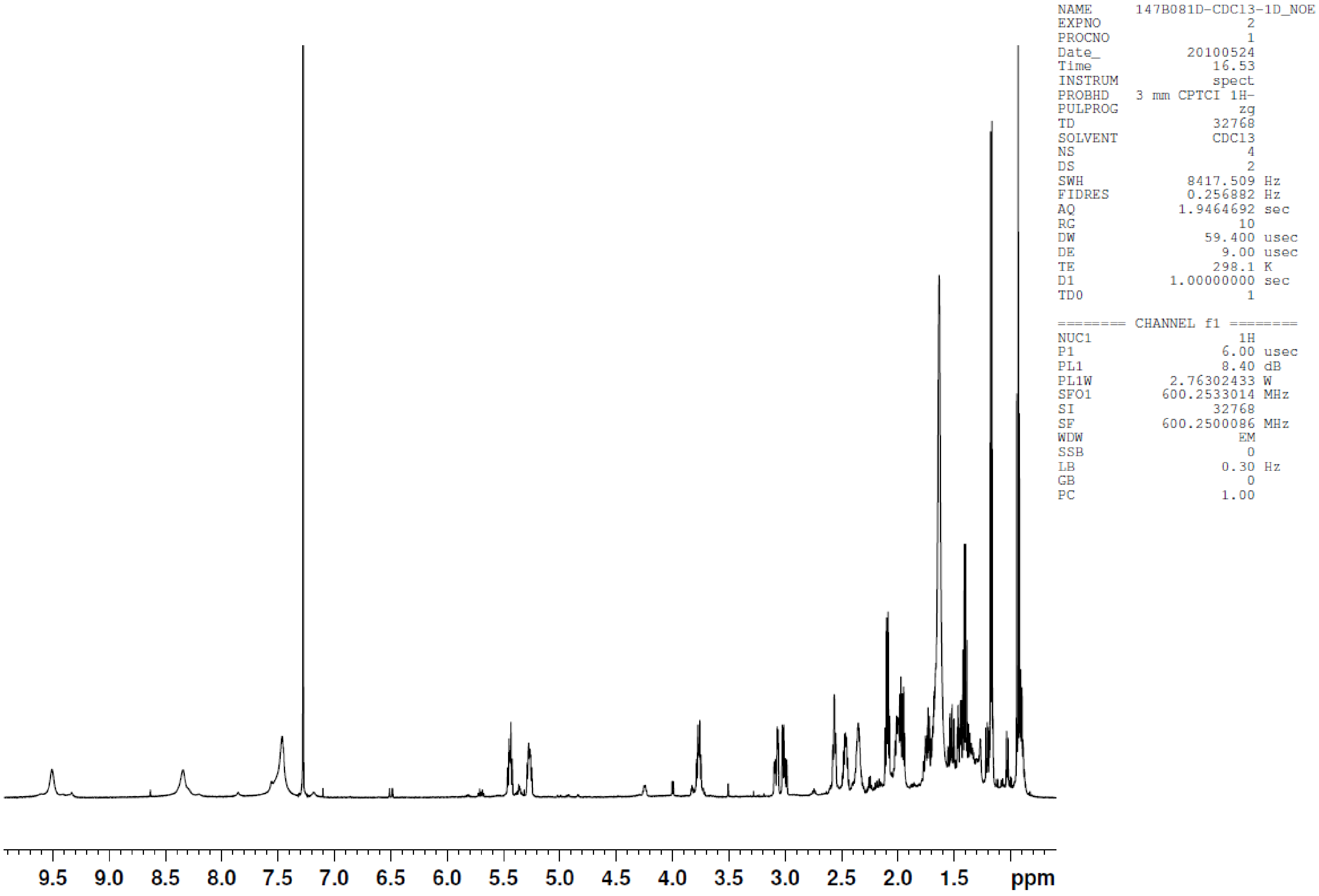

Figure S4. ^{13}C NMR spectrum of netamine M (**2**) naturally-occurring counterion in $CDCl_3$.

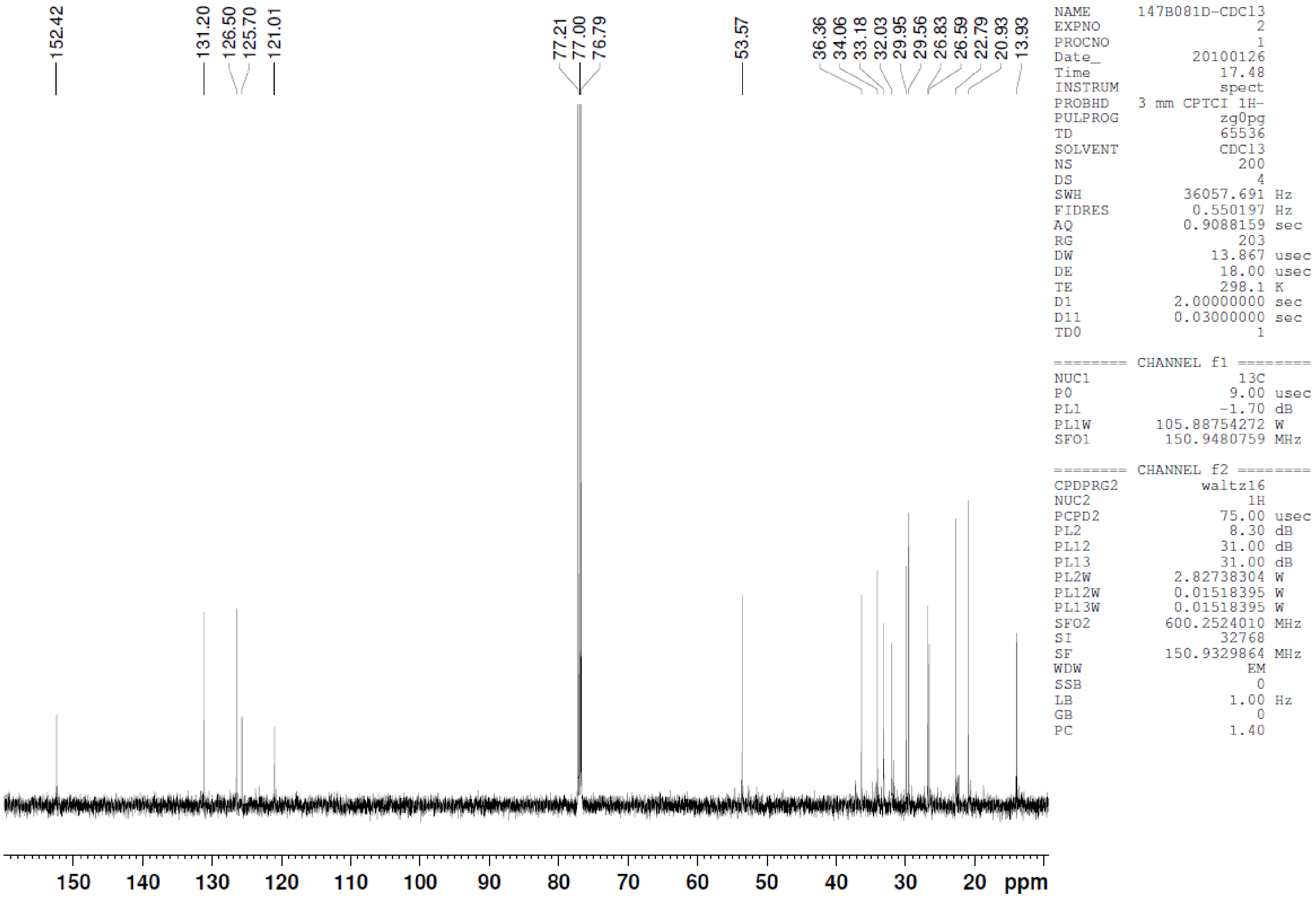

Figure S5. ^{1}H NMR spectrum of mirabilin K (**3**) trifluoroacetate salt in $CDCl_3$.

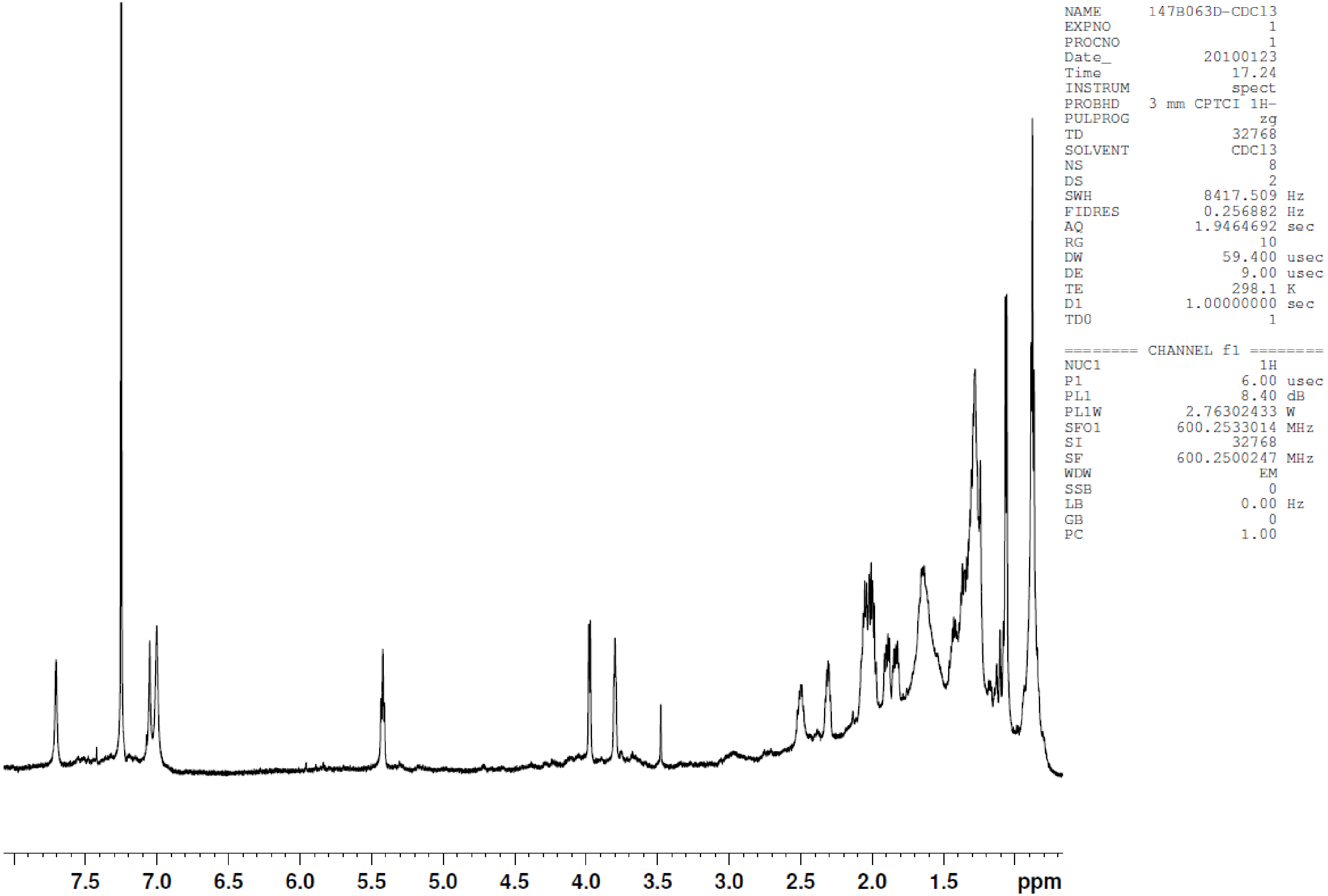

Figure S6. ^{13}C NMR spectrum of mirabilin K (**3**) trifluoroacetate salt in $CDCl_3$.

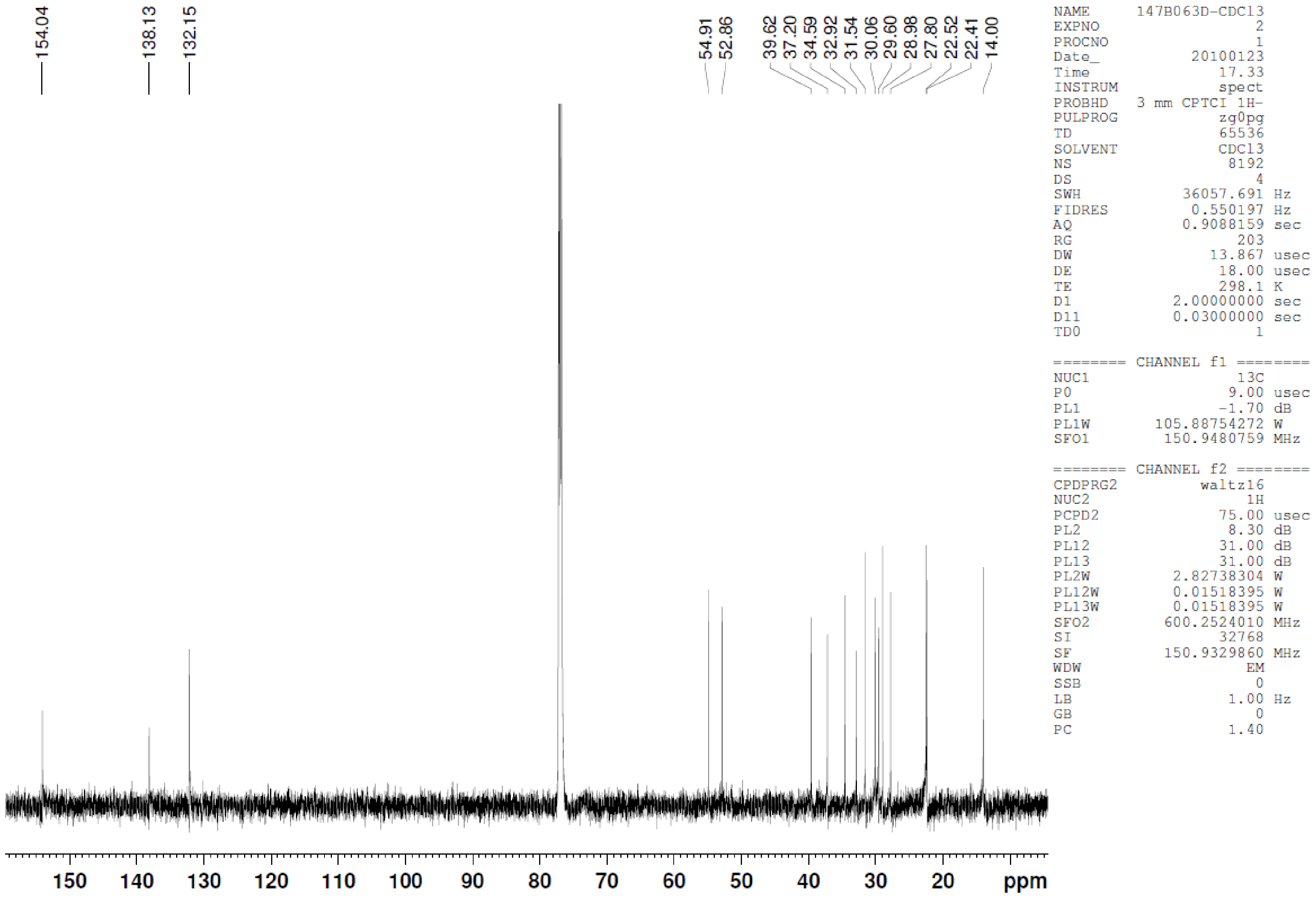

Figure S7. ROESY spectrum of mirabilin K (**3**) trifluoroacetate salt in $CDCl_3$.

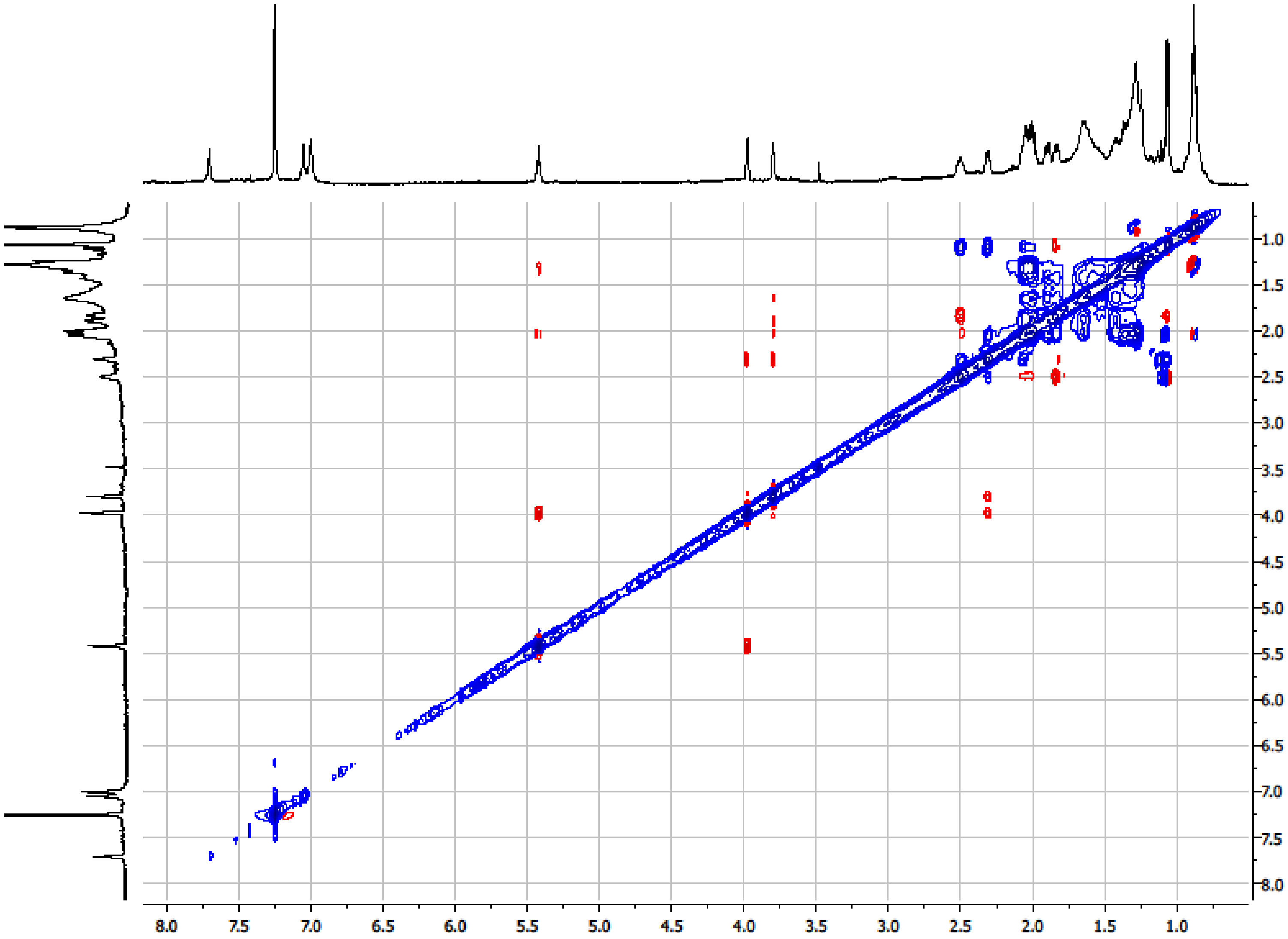

Figure S8. HRESIMS spectrum of mirabilin K (**3**) trifluoroacetate salt.

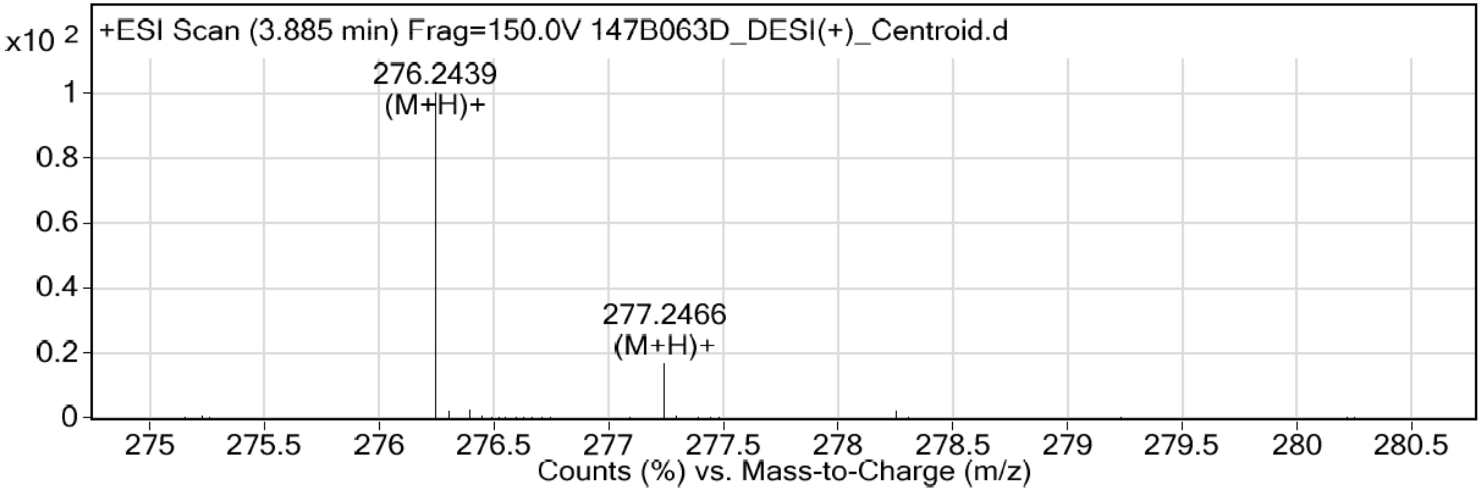

Measured Mass	Molecular Formula	Calculated Mass	ppm	DBE
276.2439	$C_{17}H_{30}N_3$	276.2434	1.8	5

Table S1. ^{1}H (600 MHz) and ^{13}C (150 MHz) data in $CDCl_3$ for mirabilin G (**1**) naturally-occurring counterion, netamine M (**2**) naturally-occurring counterion, and mirabilin K (**3**) trifluoroacetate salt.

Position	1		2		3	
	δ_C, Type	δ_H (*J* in Hz)	δ_C, Type	δ_H (*J* in Hz)	δ_C, Type	δ_H (*J* in Hz)
1-*N*		9.63 br s		9.51 br s		7.71 br s
2	152.4, C		152.4, C		154.0, C	
C2-NH_2		7.56 2H, br s		7.46 2H, br s		7.00 2H, br s
3-*N*		8.34 br s		8.35 br s		7.05 br s
4	53.4, CH	3.76 dt (10.3, 6.8)	53.6, CH	3.73 dt (10.4, 6.9)	52.9, CH	3.80 br t (3.9)
5a	32.3, CH_2	1.98 m	32.0, CH_2	1.98 m	32.9, CH_2	1.89 m
5b		1.50 m		1.49 m		1.64 m
6a	26.4, CH_2	1.72 m	26.8, CH_2	1.71 m	29.6, CH_2	1.99 m
6b				1.65 m		1.43 m
7	33.5, CH	2.45 m	34.1, CH	2.43 m	34.6, CH	2.05
8a	31.2, CH_2	1.98 m	33.2, CH_2	1.93 ddd (14.1, 6.3, 6.3)	37.2, CH_2	1.84 ddd (13.5, 5.0, 5.0)
8b		1.64 m		1.42 ddd (14.1, 4.4, 4.4)		1.10 ddd (13.5, 12.6, 12.6)
9	27.5, CH	2.71 dq (7.2, 7.3)	30.0, CH	2.32 m	30.0, CH	2.50 m
9-Me	22.5, CH_3	1.18 3H, d (7.2)	20.9, CH_3	1.14 3H, d (7.3)	22.4, CH_3	1.06 3H, d (6.8)
10	124.2, C		125.7, C		138.1, C	
11	121.1, C		121.0, C		54.9, CH	3.98 d (6.3)
12	37.2, CH	2.56 br t (6.8)	36.4, CH	2.53 br t (6.9)	39.6,CH	2.31 m
1'a	123.2	6.48 d (15.6)	26.6, CH_2	3.06 dd (15.6, 5.9)	132.2, C	5.42 br t (7.1)
1b'				2.98 dd (15.6, 8.5)		
2'	133.5, CH	5.66 dt (6.9, 15.5)	126.5, CH	5.25 m	27.8, CH_2	2.02 2H, m
3'	33.1, CH_2	2.21 2H, m	131.2, CH	5.41 m	29.6, CH_2	1.24 2H, m
4'	31.7, CH_2	1.38 2H, m	29.6, CH_2	2.06 2H, m	31.5, CH_2	1.26 2H m
5'	22.3, CH_2	1.30 2H, m	22.8, CH_2	1.37 2H, m	22.5, CH_2	1.28 2H, m
6'	14.0, CH_3	0.87 3H, t (7.2)	13.9, CH_3	0.90 3H, t (7.3)	14.0, CH_3	0.88 3H, t (6.8)

© 2014 by the authors; licensee MDPI, Basel, Switzerland. This article is an open access article distributed under the terms and conditions of the Creative Commons Attribution license (http://creativecommons.org/licenses/by/3.0/).
